# “Since his birth, I’ve always been old” the experience of being parents to children displaying disruptive behavior problems: a qualitative study

**DOI:** 10.1186/s40359-020-00465-7

**Published:** 2020-09-22

**Authors:** Britt-Marie Ljungström, Elisabeth Kenne Sarenmalm, Ulf Axberg

**Affiliations:** 1 Department of Psychology, University of Gothenburg, Box 500, 405 30 Gothenburg, Sweden; 2 Research and Development Centre, Skaraborg Hospital, 541 85 Skövde, Sweden; 3 Institute of Health and Care Sciences and Centre for Person-Centred Care, Sahlgrenska Academy at the University of Gothenburg, Box 400, 405 30 Gothenburg, Sweden; 4 Faculty of Social Studies, Department of Family Therapy and Systemic Practice, VID Specialized University, Diakonveien 12-18, 0370 Oslo, Norway

**Keywords:** Parenting, Parent training program, Parental self-efficacy, Co-parenting, Qualitative study

## Abstract

**Background:**

Being parents of children who display disruptive behavior problems (DBP) can pose several challenges. Interventions for children with DBP are primarily outpatient group parent training (PT) programs. The purpose of this study was to explore how parents of children with disruptive behavior problems, diagnosed with oppositional defiant disorder (ODD), describe the difficulties they face in their family and parenting situations.

**Methods:**

Nineteen parents of children aged 3 to 8 years who had searched for help and signed up for a parent training program provided by Child and Adolescent Mental Health Service participated in the study. Semi-structured diagnostic interviews and a modified background interview adapted for the purpose of the study were conducted before parents entered the program. All children included in the study met the DSM criteria for ODD. The interviews were audiotaped and transcribed. Thematic analysis was used to examine, identify, and report patterns of meaning in the data. The analysis was conducted inductively using a contextual approach.

**Results:**

Parents described their own vulnerability, how they were affected by the parent-child interaction, and the challenges they perceived in their parenting practices. The study contributes to an understanding of the complexity that parents of children with ODD perceive in everyday life.

**Conclusions:**

The parents in the study highlight the need to address parents’ own mental health problems, parental alliance, capacity for emotion regulation, perceived helplessness as parents, lack of parental strategies, sense of isolation, and absence of supportive social networks. All these factors could be important when tailoring interventions aimed to help and support parents of children who display DBP, and specifically ODD.

## Background

*Jim is four years old. He wants to control and decide over everyone in the family. He fights and throws things at family members. There are conflicts, several times every day. There is a word he hates and that's “no.” That’s the worst word. He does not care why they say “no”, but the word “no” has become difficult for him. The biggest problem in the family is that everything is a battle. There is a constant power struggle over small issues: whether he should sit down and eat or stand up and eat, whether he's going to drink milk or drink lemonade. There is a risk that you’ll get a fork or knife or a glass, or whatever he gets hold of, thrown in your face, or he’ll roll his chair over your feet. Big sister got a saucepan right in her face, so she had a black and blue mark on her face the last day of school.*

The quote above is an excerpt from an interview with Jim’s parents, who sought help for Jim’s disruptive behavioral problems (DBP) and were invited to participate in a parent training (PT) program. For some children there is a risk of DBP persisting and developing into severe behavioral problems or other mental illness later in life [1, 2]. For Jim and other children in similar situations, it is important that the DBP are identified in preschool years [3], so that the families can get support at an early stage. In *The Diagnostic and Statistical Manual of Mental Disorders* (DSM) [4], DBP is primarily listed as oppositional defiant disorder (ODD) and conduct disorder (CD); ODD is often considered as a disorder of early childhood and is defined mainly by an irritable disposition and resistant interactions towards authority figures [5], while CD criteria are essentially designed to describe behavioral problems in older children and adolescents [3].

There is broad agreement among researchers that the development of DBP is best understood within a transactional model in which genetic, psychological, and social factors interact [6], and that risk factors for developing DBP can be related to the individual child, family, school, and community [7]. Family context plays a crucial role in children’s development of emotional and behavioral regulation, and places great demands on parents [8, 9]. Characteristics of the child, the parents, and the interaction between parents and children appear to be more significant than other risk factors [7]. In the transactional model, mental health disorders are seen as an evolving product of reciprocal influences between children and family processes over time [6]. Not only do parents influence their children’s adjustment, but children’s characteristics and development also affect the parents in a continuous cycle of action and reaction [10]. An example of the reciprocal processes is parental depression which is considered a risk factor for children developing externalizing behavior. Children of depressed mothers have been found to have increased levels of DBP, and boys seem to be more vulnerable than girls [11]. On the other hand, when the parent–child interaction is disturbed, DBP can contribute to parent’s depressive symptoms. Research has shown a risk for parents of children with DBP to become more socially withdrawn, the parents’ relationship can become strained, and parents can feel embarrassed and stigmatized by their child’s behavior [12], which in turn can increase their burden and lead to depressive symptoms [13].

Thus, DBP in children is a complex social behavioral condition, unlikely to be caused by a single factor or circumstance, but more likely to be caused by a combination of different risk factors and high levels of stress factors [7]. As a consequence, interventions for children with DBP over the past four decades has shifted from individual child therapy in clinical settings to structured outpatient group parent training (PT) programs [14]. Most structured PT programs are based on relational and/or social learning perspectives [13] and can be a part of both selective and indicated interventions for preventing disruptive behaviors. In addition to enhancing parenting skills, such as how to follow the child’s lead, reinforce positive behavior, and effective limit setting, many PT programs also seek to strengthen parents’ self-regulation skills, particularly through increasing parents’ self-efficacy, i.e., confidence in managing the daily tasks of parenthood [15]. Associations have been found between low parental self-efficacy and children’s behavior problems; however, it is unknown whether low parental self-efficacy contributes to behavior problems or behavior problems contribute to low parental self-efficacy [16].

Even if structured PT programs have proved effective in reducing children’s behavior problems, about one-quarter to one-third of families fail to show improvement from established parenting interventions [17]. In addition to studying the effects of interventions, it is important to explore parents’ experiences of parenting a child with signs of DBP in order to better understand factors that might facilitate and hinder positive developments in families receiving PT. Therefore, the purpose of this study is to explore how parents of children displaying DBP, diagnosed with ODD, describe the difficulties they face in their family and parenting situations before entering a PT program.

## Methods

### Participants

Sixty-two parents signed up for the PT program “The Incredible Years” [18]. Some were recruited from social services or child and adolescent mental health services (CAMHS); others applied for the program after seeing notices in the newspaper or at kindergarten. Families were included in the study if the children were aged 3 to 8 years, met the DSM-IV criteria for ODD, if parents understood Swedish well enough to fill out the study forms, and if the parents provided permission for their child to participate. Parents of five children withdrew from the study. Interviews were conducted with 57 mothers and, in some cases, also with fathers or stepfathers. Nineteen of the 57 interviews were audiotaped and transcribed. This study is based on these 19 interviews. Six children were 3 to 5 years old and 13 were 6 to 8 years old; 16 (84%) were boys and three (16%) girls. All children were born in Sweden, and only one parent was not. Mean age of mothers was 33 years and fathers 39 years; 53% of mothers were married or cohabiting and 47% were separated; 11% of mothers and 14% of fathers had elementary school education only, 78% of mothers and 64% of fathers were high school graduates, and 11% of mothers and 21% of fathers had a university education; 22% of mothers and 27% of fathers were on sick leave or unemployed at the time of the interview. According to the diagnostic interview, 68% (13/19) of the children also fulfilled the criteria for Attention Deficit Hyperactivity Disorder (ADHD) beyond the ODD diagnosis, and five children fulfilled criteria for anxiety disorders (specific phobia, separation anxiety disorder and generalized disorder). Sixty-five percent of the children also met a doctor regularly because of somatic illness such as asthma, diabetes, or epilepsy. No one was excluded from the study due to neuropsychiatric disorders or other clinical problems.

### Procedure

The parents signed up for the child and adolescent mental health service’s PT program “The Incredible Years BASIC Preschool/Early School Years Parent Training Programme” (IYPP) for children aged 3–8 years, and agreed to participate in an RCT study evaluating an American PT program offered in a Scandinavian context. Written informed consent was obtained from all parents. The parents met weekly in 2 h sessions for 12–14 weeks. The IYPP is especially aimed at parents who have children with aggressive behavior, disruptive behavior problems, and ADHD. The overarching aim of the program is to reduce DBP in children by promoting a more positive interplay between children and their parents. The program strives to achieve this through interventions that improve parental function, increase parental social support, and reduce harsh and inconsistent parenting. The program involves a collaborative learning format guided by behavioral and social learning theory [18].

In order to decide whether a child met the criteria for ODD, semi-structured diagnostic face-to-face interviews were conducted with parents using the Kiddie Schedule for Affective Disorders and Schizophrenia (K-SADS) based on the fourth edition of the DSM [19]. Sixty-two of the children met the criteria for ODD and were included in the study. Parents of five children dropped out. The parents of 57 children were interviewed on two occasions - before the PT program and in a follow-up 1 year after the PT program. This study is based on the first occasion interviews. Three psychologists trained in the method of K-SADS conducted the interviews, and the first author was one of them. The K-SADS interview consists of three parts: an initial background interview with open-ended questions, a diagnostic screening interview, and a section with in-depth questions related to different DSM diagnoses. For the purpose of our research project, the K-SADS background interview was extended with questions about parenting, mental health problems, the child’s development, and related topics (Additional file 1).

At the beginning of the study, both parents were invited to participate in the interviews, but very often only the mother appeared. Later, it was decided to interview only mothers, and that is why most of the participants are mothers. All 57 interviews were documented. Parents’ answers were written down during the interview. Each interview took approximately 3 h. The interviews were also videotaped. However, after half of the interviews were completed, the research leader decided to replace the videotaping with audiotaping. In the beginning, some technical problems arose while recording, and in seven of the interviews, parts of the interview were missing. It was judged to be too complicated to transcribe the videotapes. For these reasons, the decision was made to use all the complete audiotapings that were available. Nineteen audiotaped interviews were transcribed verbatim by the first author.

### Data analysis

Thematic analysis was used to examine, identify, and report patterns of meaning (themes) in the data [20]. The analysis was conducted inductively using a contextual approach, which is considered appropriate when the aim is to describe how participants perceive their experiences. Each interview was read to get acquainted with the content. The computer program Atlas ti. 8 was used to make the huge amount of data manageable. Five interviews were completely coded first in order to identify everything of interest and relevance to parenthood. Initial coding followed the content of the transcripts very closely and produced hundreds of codes. Then a first analysis was conducted in order to organize information that was relevant to the purpose of the study. Much of the interview material described the children and their problems. Therefore, the second coding step focused on the research question, i.e., identifying descriptions and experiences of the parents. After that, the first five interviews were recoded, reducing the number of codes to less than 50. To capture the variety in the extensive data material, 14 more interviews were coded and included. During the coding process, almost no new codes emerged in interviews 16–19, indicating that there was saturation in the data. Subsequent coding was conducted to create themes and sub-themes following Braun and Clarke [20].

The first five transcribed manuscripts were read by all authors. The first author did the initial coding before meeting the other authors to discuss the codes. Together we organized the codes and information that was relevant for the study. Then the first author re-coded the five manuscripts before meeting again to discuss the coding process and sharpen the research focus. The authors continued to meet regularly during the process of analysis to discuss the codes, themes, and sub-themes in order to reach agreement on all themes and sub-themes. The decision process is illustrated in Additional file 2.

The research team is a multi-disciplined team. BML is a clinical psychologist, with a special interest in mixed methods research (MMR). EKS is a registered nurse, a director and researcher at a research and development department, and an associate professor in nursing with a main research focus on symptoms and symptom management, and in addition, has expertise in qualitative research. UA is a licensed psychologist, psychotherapist, and professor in family therapy and systemic practice with a main research focus in parenting training, parenting support, and children displaying disruptive behavior problems, thus bringing in a clinical as well as research expertise in these areas in the analysis.

### Reflexivity and ethics

The fact that two of the authors are trained clinical psychologists and psychotherapists, both with long experience from working in CAMHS in which they have met children displaying ODD and their parents, might have influenced the emerging codes and organization of sub-themes and themes. Furthermore, the education for both psychologists and psychotherapists focuses on negative and dysfunctional behaviors, which might have affected interpretations as well. However, the third member of the research team did not share the same professional experience or educational background, thus bringing in other perspectives to the coding process.

There is some evidence suggesting that depressive parents may have cognitive bias that causes them to perceive their child’s behavior as excessively negative [21]. It is therefore important to keep in mind that the descriptions of the children in this study are not always objective regarding the children’s level of functioning and personality, but might reflect the parents’ own psychosocial problems to some extent. The names of the parents and children in this text have been changed to protect their privacy. Ethics approval for this study was obtained from Gothenburg University and The Sahlgrenska Academy Ethics Committee (D: nr Ö 669–03).

## Results

The thematic analysis resulted in three main themes: “Our vulnerability as parents,” “Impact on us of the parent–child interaction,” “Challenges in our parenting practices,” and 10 sub-themes (See Table 1)

**Table 1:** Themes and sub-themes

Themes	Sub-themes
Our vulnerability as parents	Negative experiences during parents’ own childhood
	Parent mental health
	Lack of support
Impact on us of parent-child interaction	Perceptions of the child’s negative behavior and emotions
	Parent’s emotional responses to the child
	Caught in negative spirals
	Parents’ perceived helplessness
Challenges in our parenting practices	Parenting behaviors
	Parenting alliance
	Family stress

The themes and subthemes are described below together with illustrative quotes from the parents. Only pseudonyms have been used in the text. Interviews are coded as mother (M), father (F), or partner (P); parents of a girl (G) or a boy (B); and each child is assigned a number from 1 to 19. For example, a person coded *M; B3* is the mother of child 3, a boy.

### Our vulnerability as parents

Parenting is an ongoing process with roots in the parent’s own childhood. This became clear during the interviews, as parents often described their own growing up. They told about their parents and their parents’ difficulties in caring, and their absence as grandparents today. The parents also talked about their own mental health. To get an overview of the parents’ stories, three sub-themes were identified: Negative experiences in parents’ own childhood, parents’ mental health, and absence of support.

#### Negative experiences in parents’ own childhood

Several of the parents’ own upbringings were problematic in various ways. Some described growing up with parents or relatives who abused alcohol. In one family, both parents grew up with family members and relatives who abused alcohol.

*M: There are a lot of alcoholics on my mom’s side.*

*F: There are a lot of alcoholics on my mom’s side, too, so it’s the same.*

*M: But wasn’t there someone in your family—people who had depression and so on, and someone who committed suicide*? *(M and F; B6)*

Parents also described how mental illness in the family affected their upbringing. These experiences worried them during their childhood. One father had lost his father to suicide, and other parents described siblings with serious mental illness and suicide attempts. Several mothers described how their own parents suffered from anxiety and depression, as in the following quote:

*My maternal grandmother took psychiatric medication, but I don’t know for how long, and my mom has taken it since she was seventeen. Then there’s my cousin—he’s also been on medication for the past few years, and I myself have been taking antidepressants for two years now. (M; G7)*

Some of the parents didn’t grow up with their own parents. One father had to live in a boarding school from the time he was seven. His parents sent him there because of the strict discipline. During the first years, he was only allowed to meet his parents on holidays. Other parents grew up in foster families. A mother describes her ex-husband’s childhood:

*Until he was four, he lived with his mother and a lot of different men. He has never met his real dad. Then he had to move to a foster family. He had a very messy childhood. (M; B3)*

There were also stories of sexual abuse among both mothers and fathers. One of those mothers also talked about her childhood and about her effort to avoid being like her own mother. She told about her insecurity in parenting since her own mother had never been a role model for her.

*I’m the child that doesn’t exist. I’ve sat on my mom’s lap once. Once when I was fourteen, my mom, drunk, climbed onto my lap. She was married to this old guy who wasn’t my father and he was crazy. So, a lot of things happened... I myself was sexually abused. I have, like, no idea of when I’m doing something wrong and when I’m doing something right or anything. (M; B2)*

#### Parents’ mental health

While parents described mental illness in their parents, they also described their own mental health problems. Several of the mothers reported being or having been on sick leave due to exhaustion and/or depression.

*Chaos... I got no help from their father. I felt like I was going to go under. That’s when I had a breakdown. I just couldn’t take any more. (M; B13)*

*And I’ve been feeling worse and worse, you know. (M; G7)*

One father talked about his mental problems and psychiatric diagnosis. He said that he was 34 years old when he was given a diagnosis of attention deficit/hyperactivity disorder (ADHD).

*Oh, I’ve got problems myself. I’ve been diagnosed with … ADHD you know. I had a whole lot of blackouts and did a lot of stupid things that I don’t know anything about. When I get angry it’s like everything goes black, and when that happens to you, you don’t know what you’re doing. (F; B6)*

The mothers discussed the biological father’s psychiatric diagnosis more often than their own mental state, and reported that most fathers did not seek help for their psychiatric problems. This applied to both co-habiting and separated fathers. Mothers described fathers with aggressive behavior, depressive symptoms, social phobia, and symptoms of a personality disorder. Separated or divorced mothers sometimes worried about what might be happening during weekends when the children stayed with their fathers. Several mothers also stated that they had lived in violent relationships, which had significantly affected their mental state.

*It was very turbulent at that time at home, then too, so I have many gaps from when Michael was born and onwards. I don’t really remember much of it. There was so much violence and threats. (M; B19)*

Because of the father’s violence, several mothers suspected that their partners had some kind of mental disorder.

*Obviously, he must have had some kind of mental disturbance then... We went our separate ways when I was in the fifth month. He threatened me several times on the phone, saying he was going to kill me and things like that, when I was pregnant (M; B18).*

#### Lack of support

It was common that parents lacked support from their surroundings. The social networks mentioned in the interviews were of three different kinds: family, school, and professionals. Although parents expressed a strong need for support, they reported that they did not have enough. Some felt very alone in their parenting and had no one to turn to for relief:

*Well, I guess my mom has actually been sick my entire life. Off and on. I guess they have classified it very much as a psychosomatic illness. Plus, over the past few years she’s developed fibromyalgia and electrical hypersensitivity syndrome and she basically can’t tolerate exposure to anything. She is extremely... she is usually not feeling well. In my case, I guess I have felt that there’s nobody there for me because my mom can’t help me out or support me with anything. (M; G7)*

Other parents said they found it difficult to leave the child with others because of the behavioral problems:

*We’ve been child-free for one night in four years. No, there’s no one else who would dare to take on this problem. (F; B6)*

In many cases, the parents had difficulties in their contacts with preschool and schoolteachers. The parents wanted much more support from the teachers. One mother wanted the teacher to help her child go to the bathroom because without help he would have a bowel movement in his pants. Some parents wanted the child to be tested by a psychologist, because behavior problems need a diagnose before funds are granted to adapt the school environment. Other parents found it difficult getting support, if the child didn’t have a special diagnosis.

*I feel that if you don’t have a document stating that you’ve got certain problems, it’s difficult for a parent to assert that Jesper might need... The way I see it, Jesper needs a little extra support in school, but it’s very hard to get recognition for that. (M; B1)*

Parents asked for more support and advice from teachers, but did not feel heard in their attempts to communicate.

*So, of course, I got no support from them [the school] either. Instead, I was supposed to change my behavior. How am I supposed to change my behavior when I am not told how, or what, or why? (M; B10)*

Others felt that contact and communication with the teachers was very sparse.

*Of course, we know nothing about how things are at school... since we haven’t had any follow-up interview or anything yet, so I don’t know. And I almost never see the teacher and there’s always so much stress—she doesn’t have time. (M; B13)*

Many families also had regular contact with healthcare and social services professionals. Some parents explained that professionals didn’t seem to take their worries seriously, which therefore delayed efforts for the child. Other parents said that a major problem was being sent to different physicians and different care facilities, with no one really taking responsibility for helping the child:

*Because I have felt I’m not getting the right kind of support at the pediatric clinic. Instead, we’ve just been bounced back and forth between the asthma doctor and the epilepsy doctor and so on.... Because we kind of felt it couldn’t go on like this. And you just collapse completely, you know, it doesn’t work in the long run, and he’s getting bigger and bigger. He’s going to start school in the fall and we kind of want him to have the right start*. *(M; B11)*

### Impact on us of the parent–child interaction

Defiant behavior was described as occurring in the parent–child interaction. The parents described not only their perceptions of their child’s behavior and emotional expression, but also their own behaviors and feelings toward the child and what happens in the relationship. Four different sub-themes show this interaction. The first addresses parents’ perceptions of their child’s negative behavior and emotional expression. Sub-theme two focuses on parents’ emotional responses to their child, sub-theme three describes negative spirals in the interaction, and the last highlights parents’ perceived helplessness.

#### Perceptions of the child’s negative behavior and emotions

The parents mainly talked about four different types of their children’s behavior and emotions: aggression, self-sufficiency, controlling behavior, and unpredictability. (See the process of quotes, codes and the sub-category in Table 2 in Additional file 2). The most common descriptions were of aggression and unpredictability. Parents expressed the strong feeling that the child’s aggression was directed especially toward them.

*Plus, these outbursts are actually mainly focused on me. (M; G7)*

*And it’s sickening the things that come flying out of his mouth—he says “I’m gonna kill you!” (M; B18)*

Some children screamed and threatened that they would destroy the house or kill the parents, and a few parents experienced the child’s aggression as both calculated and threatening. Parents also described an escalation from verbal to physical threats. One boy threw cutlery and glasses at his parents and siblings*.* Other children spat, kicked, and some used objects as weapons.

*I don’t remember how the quarrel started, but suddenly he was going to... he said he was going to kill me. And then he said he was going to kill his sister, too, and she’s sitting right there listening, and then he said he was going to kill me first, because if he killed her first I’d have time to call for help, so he was going to kill me first because she wouldn’t have time to call for help because she isn’t as quick as I am. And of course… and so you think... (M; B1)*

Other parents perceived their children as withdrawn, not seeking, or even rejecting the parents’ emotional support. These parents described their grief over not being able to hug or be close to their child.

*He has difficulty relating to adults. He has an easier time with kids, but he doesn’t let adults get close to him... he doesn’t trust them... he doesn’t ask for help. He’s not the kind that will crawl up onto your lap and, you know, want to be comforted. Instead, if he’s upset about something, he goes off by himself. (M; B1)*

*He wouldn’t let you touch him, wouldn’t let you pick him up and hold him and comfort him. (F; B11)*

Some parents found themselves controlled by their child, who exerted pressure on the parent by crying, screaming, or playing helpless.

*Well, she screams, “You don’t love me. I’m not putting that sweater on because you don’t love me!” This can go on for an hour. If she doesn’t get what she wants, she can lie down on the floor and scream for an hour, basically until she falls asleep. (M; G9)*

Often, the mothers felt that there were power struggles between parent and child.

*So, you know, it’s a power struggle between her and me. And I even feel sometimes in the morning, “I wonder how this morning is going to start out!” (M; G4)*

A great concern for parents was the unpredictability of their relationship with the child. This was described mainly in terms of emotional and behavioral unpredictability. Parents explained that their child’s emotional state could change so quickly that they sometimes did not understand what had happened, and they could never predict or prepare for when it might “break loose.”

*Sometimes I’m amazed at how such small things can trigger such huge outbursts of rage. Because sometimes things happen where I’m expecting a scene… and yes, there is indeed a reaction, but not very strong. And in some way that, too, creates uncertainty: you never really know what’s coming. (M; B1)*

They also described how their child’s reactions could swing rapidly back and forth between positive and negative emotional expressions.

*And then there’s the fact that it changes so quickly. You are still feeling rage and for her there’s been a change. Then suddenly she starts talking about something else and she’s chirping away like a happy little bird. (M; G4)*

Parents said they could never be sure of how the child would behave in different situations, which created fear and uncertainty in relation to other people, for example, when going shopping, visiting relatives, or taking a trip.

*Should we take Jim shopping with us? Will he get mad? And if he gets mad, he grabs the shopping cart and rams it into people. Will he run away? Will he run out of the store? It’s different every time. (F; B6)*

A mother also described the uncertainty she felt when her son was in school.

*It might be about some little thing, or it could be that he’s angry at someone coming up and touching him. Then he explodes... it’s like he’s got demons inside of him—his eyes roll around and he stands there saying “I’m gonna kill the teacher!” and it frightens the other kids. He grabs everything on the shelves and drags everything down. (M; B18)*

#### Parents’ emotional responses to the child

The parents reported very strong emotions in their interaction with the child, including difficulties controlling their anger when the child was challenging. Some parents felt extremely provoked by their child’s behaviors, and others felt sad or even afraid. Someone said that they had stopped caring at all, and a couple of mothers admitted emotionally rejecting the child. Common emotional responses to the children’s difficult behaviors were anger and frustration.

*I mean I can’t come along and be nice and try to bargain, and I can’t stand there yelling and screaming. I can’t, like, practically pummel the life out of him, because nothing helps. So, I never hit my son, but there are times when I feel like walloping him because he doesn’t hear anything. And nothing helps. (M; B10)*

The parents described how strong feelings of uncontrollable anger arose in the interaction with the child.

*He has to do it eventually, but then it ends with me exploding and going crazy, and I hate that because I don’t think things should go that far, you know? (M; B2)*

Some parents felt provoked by the child’s inability to, for example, speed up in the morning or finish a computer game, but did not feel the child provoked them intentionally. However, other parents felt that the child challenged them and were extremely provoked by their child’s behavior.

*Well, he can sit and jeer at me when we get into arguments, I mean when I try to physically force him to take his pants off, because he refuses, and I simply can’t take it. I don’t manage to get the pants off him. He laughs at me and it drives me insane. I don’t know what to do, because he wins, because I simply cannot manage to get the pants off him. I am too weak. It has happened a couple of times that he’s mocked me, saying “You couldn’t get them off!” and it’s such a sickening feeling! (M; B12)*

Some of the mothers described how sad and hurt they felt when their child struck them, and how complex emotions about the child arose in them, which led them to react by pushing the child away.

*It’s deeply painful, even though the blow itself doesn’t hurt. So, it hurts me... And there are times when I feel sorry for Judy [the daughter], but there are also times when she has been, you know, extremely mean, and I have felt hurt and then she wants me to comfort her. At those times I push her away, because I just can’t do it then. And of course, obviously that hurts her, too, but she doesn’t say it. Or indicate that she feels hurt. (M; G4)*

#### Caught in negative spirals

Sometimes parents reported negative spirals in the interaction with the child. The parents’ efforts to constrain the child escalated the child’s behavior, which made the parents even angrier and escalated the child’s behavior further.

*I mean sometimes it’s simply not possible to stop him—it just doesn’t work. He keeps on getting more and more irritated, he keeps on screaming and hitting and threatening to kill me, destroy the building, he’s gonna—I mean, it’s too much—you have to grab him and carry him up to his room. So, he lies there, screaming and throwing things, and if you just leave him to lie there, he suddenly starts crying and being sad. Then maybe he comes out and starts testing you again... but then it’s back to the room, and like, “Now you better... You cannot behave like this.” (M; B12)*

Sometimes it could take a couple of hours before the child (and the parents) calmed down.

*He never gives in... he’s on me, bugging me and bugging me, so it’s enough to drive me nuts. So, these fights can go on for anything from half an hour to up to two hours*. *(M; B2)*

#### Parents’ perceived helplessness

Parents described chaotic situations in the family. They did not understand their child. They realized that something was wrong but could not really put it into words.

*As I said, when Thea [the daughter] isn’t home, “It’s so calm,” “It’s so pleasant,” and that’s of course not how it’s supposed to be. As I try to answer these questions, I’m finding it difficult to put my finger on just what it is. I mean, there’s something—presumably there’s something inside of her that she’s not getting out. That we can’t quite get a grip on. (M; G9)*

Parents had many thoughts and questions about the quality of their own parenting and why the children behaved the way they did.

*And so that’s where I feel we’re unable to cope. So much with Ethan [the son] is really our own fault—we don’t know what to do. (M; B15)*

*No, I want to know if there’s something wrong—if he needs help, or if I need help? Or if it’s just ordinary rebelliousness or if it’s normal for this age? I can’t figure it out. I need help. (M; B3)*

Parents explained it was hard to feel they did not understand their child and they also realized that they did not have the tools to cope with their child’s challenging behavior. They experienced very many situations when they felt helpless and incapable in their parenting.

*That I feel powerless. I feel like I’ve lost this. (M; G4)*

*Because every morning is a chaotic situation and I don’t know what to do. (M; B12)*

### Challenges in our parenting practices

During the interviews, participants described some common things in their parental roles as major challenges. The parents had obvious difficulties raising their children and greatly needed an expanded repertoire of strategies. They also had problems maintaining a strong parenting alliance and were also stressed by the child’s externalizing behavior and its consequences on the rest of the family.

#### Parenting behaviors

Two main strategies are discernible in the parents’ descriptions of their parenting behaviors. They described themselves either as controlling parents or as more passive. Some parents became authoritarian, punitive, threatening, or nagging in order to maintain a sense of control. Their most common way of dealing with the child’s externalizing behavior was with some kind of physical boundaries.

*For some time now we’ve had this routine where we have a chair and if you’re not behaving yourself you have to go and sit in that chair until you say you’re sorry. So, he sits there, like this, but he sits still, because he knows he’s not allowed to leave the chair. (M; B13)*

Some parents felt forced to carry the child to their room, while others said they used physical punishment in difficult situations

*Little sister—it’s impossible. She refuses to go up to her room. You have to carry her up and then she’s been kicking... and she gets really angry and red in the face. (M; G4)*

*Okay, I’ve grabbed him by the ear, and he’s been spanked now and then if he’s gone too far, right? (M; B2)*

Some of the mothers said that they incessantly nagged the children to make them obey.

*But I think that since it’s mainly been me who’s been at home with him, who’s brought him up all the time, and who’s always been the one telling him off.… Oh, every day. I have to get after him all the time. So, I imagine he thinks I nag him a lot. (M; B8)*

Other parents described a more passive approach to their children. They did not take a governing role, but rather let the children take control. One common cause for this attitude was that the parents were unable to cope with the conflicts.

*Well, he behaves worse with me. Because he knows I give in more easily... because I don’t have the energy to deal with the consequences or the problem by myself. (F; B6)*

Other reasons for a passive approach were parents’ feeling unable to limit the child or thinking it would be more peaceful and quiet at home if they avoided conflicts.

*It’s so bloody hard for me to set boundaries. I’m such a hopeless kind person. I can’t be angry at anyone. I can’t, like, yell—I can’t get angry at anyone. I simply cannot be stern. That, I believe, is a large part of why Otto is the way he is; I’ve never set real boundaries. I often give in, since I cannot get angry, even though I might be thinking to myself “Why the heck did I give in?” And then I might say “No more computer for you,” “No way,” he says then, and “I’ll get to play on the computer anyway, I know.” (M; B18)*

A group of parents tried but found it hard to be consistent in their parenting behaviors, usually because they did not have enough energy.

*But I try to be consistent, but at the same time I am alone, so it’s hard to be consistent all the time, because I just don’t have the energy. (M; B10)*

#### Parenting alliance

It was a challenge for parents to maintain a uniform line in their approach to the child. Many parents reported that they were often in disagreement and described the consequences of their conflicts. This section contains descriptions of how parents allied in their attitude toward the child, and whether they had the same or different ideas about raising and responding to the child. About half of the participating parents were cohabiting or married, and most who were separated shared custody of the child. Therefore, they needed to cooperate with the other parent. In the interviews, it was much more common for parents to report disagreement than agreement on the child’s upbringing. In the group of cohabiting parents, the disagreements seemed to arise mostly when one partner found the other to be either too kind and reluctant to deal with the child, or too authoritarian and strict.

*I’m more controlling, and I look for more order and routines. He is more relaxed, you might say, and lets things go the way they go, sort of. (M; G7)*

Often the parents agreed that the child had problem with disruptive behavior, but did not agree on how to deal with and respond to the child. In some cases, the disagreement could lead to strong conflicts between the parents, and they felt that the child used the situation to their own advantage.

*He [Ethan] plays us off against one another, I have to admit. There are certain situations in which neither John nor I know what we should do, and there are times when I get angry at John if I think John is being too harsh and getting too angry, and why should Ethan have to go to his room just then? It often ends with us being on the outs. And then Ethan has got his way. (M; B15)*

In the case of parents who were separated, disagreements fell mainly into three categories: whether or not the child even had problematic behaviors; what routines the child should follow (often resulting in different routines for the child with each parent); and inter-parental conflicts so strong that the parents had no communication at all.

*There are no real routines at dad’s place, and we note that showering is not working out either. He’s wearing the same underwear that I sent him off in. Those routines aren’t enforced at dad’s place—everything is just about having fun. (M; B12)*

Separated mothers also had disagreements with a new cohabiting partner about the attitude toward the child. Mothers thought it was easier for their partner to be hard and consistent with the mother’s child than with his own children.

*Then again, I think it’s easier to be more consistent with the one who is not one’s own. We don’t always have the same opinions, and unfortunately, it’s not unusual that we have... we give different messages in one and the same situation. I might say one thing and he says another, and vice versa. (M; B13)*

The mother above said it was very positive to live next door to her parents, but she also realized that it was a problem when it came to mutual strategies in dealing with the boy, who challenged his environment quite vigorously.

*In these situations, it can be that, since we live so close to grandma and grandpa, they have one way of handling him. Andrews [stepfather] has one way of handling him, I have one way, and [Tristan’s] father has another way. So there may be four different child-rearing methods being applied in the same area. (M; B13)*

#### Family stress

The families lived under stress mainly related to four areas: concern about neglecting the needs of the other siblings in the family; the difficulties bringing the child into social contexts; constant monitoring; and the great energy required to manage the child’s behavior problems. Having children with ODD affected other children in the families. The parents felt insufficient in caring for the others and were worried that the other children felt neglected and often had to stand back.

*Well, it’s like we’ve always said: if he’d been the only child, then okay, but we have three other children and they have needs, too. They take his needs into consideration, but he shows no consideration for anything. Because he’s the only one who needs anything. (M; B6)*

It was very common among the families that parents worried a lot about bringing the child into different social contexts. Many times, they chose to stay home, which contributed to increasing the family’s isolation and loss of social networks.

*Well, sometimes we have almost structured our entire life around him. He has totally steered everything…like we have tried, we try to make it so his life is simple, right? We don’t come up with anything special (F; B11)*

*For example, we don’t go away on any trips. Occasionally we get invited to visit our parents’ homes or to have dinner with someone, but we generally don’t go. Because it is so stressful for him. (M; B3)*

The parents explained that there was always a need to be one step ahead in everything that was going to happen in the family, and always had to prepare the child for all planned activities. Therefore, there was a need for constant monitoring. The parents did not dare leave the child with younger siblings, in sports activities or with friends.

*Constant monitoring, constant monitoring. If I just go to pee, I’ll take the younger siblings with me. (M; B19)*

*We feel that we have a child that requires monitoring twenty-four hours a day, in every situation. (M; B1)*

The parents also described the strain they experienced in having a child who had externalizing behavior. The parents experienced aggressive outbursts as especially burdensome.

*Well, it’s the rages. They are hard to take. It’s hard to admit that you find it difficult. It goes up and down, and she’s certainly calmer, but then it gets worse and you start to think you can’t take it any more. (M; G9)*

Other stressors from the children were defiant behaviors, hyperactivity, and difficulties unwinding in the evening, which made it difficult for parents to recover from the day.

*Should I talk about how hard it is? It is so hard that I hear him in my mind every night as I get ready to go to bed. (M; B6)*

*Given that he was showing ADHD symptoms even as an infant, I became pretty old pretty quickly. Since he was born, I’ve always been old. (M; B10)*

## Discussion

There are a large number of studies that have examined the efficacy of PT programs [13, 17, 22], but less is understood about which aspects in PT programs that make interventions useful and meaningful for the families [23]. Even fewer studies describe the parents’ own experiences of having a child with DBP. It is important to be aware of these experiences and to take them into account in order to help understand which factors might facilitate or hinder positive developments in families receiving PT [22]. This study aimed to describe how parents experience the difficulties of parenting children who fulfill the criteria for ODD. These descriptions are captured before the parents started their participation in the Incredible Years PT program provided by a child and adolescent mental health service in Sweden. A thematic analysis was carried out, and the analysis of the interviews reveals the outer and inner complexity that parents perceive in their everyday life. Three main themes were developed from the analysis of the interviews: the parents’ own vulnerability, how the parents were affected by the parent–child interaction, and the challenges they perceived in their parenting practices.

Several parents spontaneously discussed their own upbringing and described mental health problems among their family members, while others talked openly about other negative experiences like childhood sexual abuse they had experienced, growing up in foster care, or growing up with parents who abused alcohol. These worrying experiences from their own childhood, their family history, and how emotions were expressed in their family of origin, all affected how they interacted with their own children, which is in line with Christie et al., [24]. It seemed as though a pattern of mental ill-being was reproduced through the generations as participants described their parents’ mental health problems, their own, and their children’s. Mental health problems make parenting, already difficult, much more challenging. Parents in this study did not seem to seek help for their mental health problems. Researchers have developed a growing interest in examining changes in parents’ mental outcomes after participating in PT programs. The results of such studies are mixed. Barlow et al. [25] found that PT programs can be effective in improving maternal depression, anxiety, and stress in the short term, while Leijten et al. [13] found improvements in psychosocial health only in cases where there was severe maternal depression in combination with severe child disruptive problems. However, from the children’s perspective, it is vital that parents with mental health problems seek help to improve their parenting ability, so that the children’s development is not adversely affected. It is also important to consider parents’ mental health problems when planning family interventions. However, research shows that PT programs tend to be less effective if combined with other efforts at the same time. Therefore, timing is important in implementing efforts to improve parents’ mental health [26].

Parents in the current study also expressed feelings of loneliness and isolation. The African proverb “It takes a village to raise a child” means that it requires interactions with an entire community of people for a child to thrive and grow in a safe environment. This thought is very applicable to families of children with ODD, who are particularly dependent on good and trusting relationships with various networks. Two-thirds of the children in this study had somatic diseases along with their behavior problems, which meant they had many healthcare meetings with physicians and nurses, as well as contacts with school and social services. Parents felt alone and often misunderstood in these contacts, and felt that no one really took responsibility. Unfortunately, it was also common for these parents to lack important support from close family members such as grandparents. PT programs that involve meetings with other people in the family’s social networks can reduce the parents’ stress by helping these other important people to better understand the situation. Furthermore, the PT program often becomes very important to the parents. Participation breaks their isolation and allows them, maybe for the first time, to meet other parents with similar problems [27].

What is particularly evident from the parents’ descriptions is that regulation of the children’s behavior and emotions takes place in a context that is problematic. Four different emotionally strenuous experiences were clearly visible in the interactions: the child’s behavior with the parent, the parents’ feelings when interacting with the child, negative spirals in the parent–child interaction, and the parents’ strong feelings of perceived helplessness. In summary, parents and children often ended up in destructive interactions and confrontations with each other, where both parties had difficulty regulating their behaviors and emotions. Crespo el al [28]. assumed that parents’ overall style of emotional regulation impacts their regulation in the parenting context, and exposure to the parent’s pattern of regulation and behavior directly impacts the child’s emotional reactions and regulation. However, we should not forget that the parent–child relationship is by nature bi-directional and transactional [16]. Children with difficult temperaments place increased stress on parents’ emotion-regulation capacities [28]. Parents with difficulty regulating their own emotions might struggle more to cope effectively when their child expresses negative emotions. Parents’ awareness of their own emotions and internal distress plays a crucial role in how they respond to the child in stressful situations. Parents who are not aware of their own emotions may have difficulty supporting their children’s developing emotional understanding. They may serve as poor external regulators in emotional conflict situations and fail to teach the child coping and regulating strategies [28]. Therefore, it seems extra important that parents gain insight into their own emotional reactivity and learn how to respond calmly toward their child. Considering the regulatory difficulties described by several of the parents, both in themselves and in their children, emotional regulation components might be crucial in interventions for parents of children displaying ODD. For example, in the IYPP program, the parents are trained in “self-talk” in which they can encourage themselves to calm down and use positive strategies to manage a difficult situation.

Many parents also described intense escalations of parent–child aggression, which led to chaotic situations in the family. Bi-directional difficulties with regulation can lead to a coercive pattern between the child and the parents [29, 30], characterized by lack of positive interactions, ineffective and inconsistent limits on the child’s behavior, and a reinforcing spiral of scolding and escalated child’s behavior, eventually leading to the parent withdrawing. These negative spirals can leave parents with a sense of lost authority and helplessness. PT programs based on social learning principles aim to strengthen parental self-efficacy [31]. Awareness of one’s own inner strength is associated with increased skills in parenting and promotes a greater sense of control and parental self-efficacy [32]. Parents with low parental self-efficacy perceive their children as having a multitude of behavior problems, while parents with high parental self-efficacy demonstrate more effective parenting even in the face of challenging child behavior [16]. For those parents who experience negative emotional spirals with their children, it seems important to be supported in moving from perceived helplessness to a greater sense of perceived parental self-efficacy throughout the PT program [29].

The parents participating in this study, both co-habiting and separated, told stories of how difficult it was for them to have common strategies towards their children with DBP, and how difficult it was to maintain a good alliance with each other. An essential part of raising children together, especially with children who are more challenging than most, is to have mutual strategies and compatible parenting styles [33]. A poor co-parenting alliance influences the family system’s adjustment and increases parenting stress [34]. In their evaluation of “The Incredible Years”, Axberg & Broberg [35] found that perceived parenting alliance increased more in the group who participated in the PT program than in the control group. They concluded that parents who participated together and learned to support each other in parenting, strengthened their parenting alliance. However, one-fifth of the mothers participated without a partner [35]. Therefore, it would be beneficial if clinicians encourage parents to participate together in the PT program as far as possible, even if the parents are separated.

Many of the families in our study described a variety of stressors in their lives. The stress was apparent on several levels: individually and in relation to the family of origin, the other parent, the child, the preschool or school, and other actors in the social network. Parents made many adjustments to reduce stress in their families but were extremely tired. The challenge of constantly dealing with disruptive behavior was a great burden. It is important to realize that parental stress may also be due to a sense of failing as a parent [36]. One important result of this study has been to highlight descriptions of both the inner and outer complexity of families of children with ODD, in order to avoid placing more unnecessary blame on the parents. If not attended to, there is a risk that participation in a PT program might sometimes increase parental stress, which in turn can increase feelings of guilt and failure in parenting, reduce the experience of parental efficacy, and thus counteract the desired effects of intentions with a PT program [22, 36].

PT programs, especially those based on social learning theories like IYPP, contain components to reduce coercive patterns in the parent-child relation. Many of those PT programs have the ambition of teaching parent emotional regulation strategies and flexible parenting strategies to reduce the feeling of helplessness and to increase parental efficacy. On the other hand, PT programs with a relational focus emphasize parental awareness, understanding and acceptance of the child’s feelings, where the aim is to promote a positive development towards autonomy in the child. When children are at risk developing long term DBP, this study has shown that the parents need support based on both of these perspectives in parenting in order to cope. They need to cope with the challenges of parenting a child with DBD, while still being a responsive and loving parent that also sets clear boundaries. We also want to emphasize the importance of careful assessments before parents join the PT program in order to identify the family’s unique needs. This would probably lead to a greater understanding and a greater empathy with the parents’ different difficulties. The current findings suggest that tailoring program content to meet individual needs of families is of particular importance for the parents, even if it poses a challenge to practitioners delivering structured programs following specific manuals. It can be difficult to balance fidelity to the manual and flexibility towards the participants. Furthermore, for those families with particularly destructive interactions between child and parent, as was seen in some cases in this study, we suggest that practitioners should also consider offering interaction therapy options, since the relation between parent and child is crucial.

### Limitations

The contributions of the present study should be considered alongside its limitations. The desire to describe the breadth of the parents’ difficulties has been carried out at the expense of depth in the analysis. As with all qualitative research, caution should also be taken when generalizing the study findings. Participants in this sample are not representative of every family of children with ODD. Nevertheless, the study highlights difficulties faced by 19 different families who have children with clinical levels of ODD hoping to participate in a PT program. The participants included 19 mothers and 3 fathers/stepfathers. However, it would have been interesting to give more voice to fathers in the study. For future research, it is important to investigate more systematically which stress factors or combinations of stress factors contribute to the inability of many families, often 25 to 30%, to recover after a PT program.

## Conclusions

The results of this study may contribute information to clinical practice, especially for clinicians working with prevention programs for parents’ who have children with disruptive behavior problems. This study highlights stressors that might affect the parenting interventions negatively. Above all, the parents in the present study show the need to address parents’ own mental health problems, their emotional regulation capacities, the parental alliance, their perceived helplessness in the parental role, a lack of parenting strategies, and their sense of isolation and lack of supportive social networks. All of these could be important in tailoring interventions to help and support parents of children who display DBP, especially ODD.

## Supplementary information

**Additional file 1.** Additional questions in the K-SADS background interview.

**Additional file 2: Table 2.** Description of theme (2) sub-theme (1) and codes with supporting quotes.

## Data Availability

The dataset collected and analyzed during the current study are not publicly available as this could compromise participants privacy. The corresponding author can be contacted with questions considering the dataset.

## Abbreviations

ADHD: Attention deficit hyperactivity disorder
CAMHS: Child and adolescent mental health services
DBP: Disruptive behavior problems
ODD: Oppositional defiant disorder
PT: Parent training
