# Supplementary material for: “Since his birth, I’ve always been old” the experience of being parents to children displaying disruptive behavior problems: a qualitative study
Source: BMC Psychol. 2020 Sep 22;8:100. doi: 10.1186/s40359-020-00465-7 (PMC7510140; doi:10.1186/s40359-020-00465-7)
Supplement: Supplementary file 1 — Additional file 1. Additional questions in the K-SADS background interview. [file 40359_2020_465_MOESM1_ESM.docx]

**Appendix 1.**

**Additional questions in the K-SADS background interview.**

**A. General questions**

1. Are there siblings to the child in the family? Ages?

2. How old is the identified child?

3. Is there any other caring person in the social network? Who is that?

**B. General life situation**

1. Describe the situation of the child. Where does the child live? Separated parents? When did the parents separated? Shared custody? How often do the child lives with the other parent?

2. Age of mother, age of father?

3. Education of mother and father? Working place? On sick leave? Unemployment?

4. Are the parents native?

**C. Development history**

1. Complications during pregnancy or childbirth?

2. When was the baby born? In expected time?

3. Describe the child during infant and toddler period. (Sleeping, eating, temperament...)

4.The child´s ability so socialize during infant and toddler period?

5. Milestones in development within normal limits?

6. Has the child visit doctors due to illness or accident? Has the child stayed at hospital? For how long time, how often?

7. Has something related to visits at hospital or in the contact with child health services been troublesome?

8. Have the child been separated from you more than a couple of days for other reason than being at hospital?

9. Does your child has experienced parents´ death, death of older siblings, death of younger siblings, serious illness in any family member?

**D. The problems and their context**

1. Short description of how the child functions at home with parents and siblings.

2. Short description of how the child functions in preschool or school.

3. Short description of how the child functions with peers.

4. Describe the problems that caused you to contact us.

5. Describe the (three) major problems you experience with your child.

6. When did the problems started?

7. In which environments is it most problematic?

8. Have you had or have you now any contact with any professionals for the problems? What problems were these? What kind of treatment did you get? The results of the treatment?

9. Does your child get any medical treatment now, medicines?

10. With how many different professionals have you had contact?

11. Are there anyone else in the family who has or has had similar problems?

12. Are there anyone else in the family or other close relatives who has psychiatric problems or substance abuse?

13. How do you as parents agree on how problematic it is with your child?

14. How do you as parents agree on what to do and how to treat the child?

15. Describe your way on raising the child.

16. Describe the other parent´s way of raising the child.

**E. The current situation**

1. How did you get in contact with us?

2. Does your child go to preschool or have any child minder?

3. Does your child participate in any leisure activities? What kind?

4. Has there been any problem in preschool, school or in the leisure activities`

5. How many close peers does your child has?

6. How often does your child meet with his/her peers?

7. Describe your child´s good and strong qualities.
