# Supplementary material for: “Since his birth, I’ve always been old” the experience of being parents to children displaying disruptive behavior problems: a qualitative study
Source: BMC Psychol. 2020 Sep 22;8:100. doi: 10.1186/s40359-020-00465-7 (PMC7510140; doi:10.1186/s40359-020-00465-7)
Supplement: Supplementary file 2 — Additional file 2: Table 2. Description of theme (2) sub-theme (1) and codes with supporting quotes. [file 40359_2020_465_MOESM2_ESM.docx]

**Table 2** Description of theme 2. sub-theme 1. and codes with supporting quotes

| Theme 2. | Sub-theme 1. | Codes (4)** | Quotes in sub-theme 1. (60)** |
| --- | --- | --- | --- |
| Impact of us of parent-child interaction | Percep-tions of the child´s negative behavior and emotions | Unpredic-table | **It might be about some little thing, or it could be that he’s angry at someone coming up and touching him. Then he explodes... it’s like he’s got demons inside of him—his eyes roll around and he stands there saying “I’m gonna kill the teacher!” and it frightens the other kids. He grabs everything on the shelves and drags everything down. (M; B18)* |
|  |  |  | *If he is in a good mood, he can take almost anything, but you never know when he loses it. (B; M6)* |
|  |  |  | **Sometimes I´m amazed at how such small things can trigger such huge outbursts of rage. Because sometimes things happens where I´m expecting a scene…and yes, there is indeed a reaction, but not very strong. And in some way that, too, creates uncertainity: you never really know what´s coming. (M; B1)* |
|  |  | Aggressive | **I don’t remember how the quarrel started, but suddenly he was going to... he said he was going to kill me. And then he said he was going to kill his sister, too, and she’s sitting right there listening, and then he said he was going to kill me first, because if he killed her first I’d have time to call for help, so he was going to kill me first because she wouldn’t have time to call for help because she isn’t as quick as I am. And of course… and so you think... (M; B1)* |
|  |  |  | *Threatens us and… he has hit you (turning to the mother) a couple of times with things. He has hit me a couple of times with things too. I mean with sticks and stuff. He talks a lot about using weapons. Yeah, he does talk quite a lot about using weapons and hurting someone. (P; B12)* |
|  |  |  | *Yeah, because she becomes physically aggressive. When I for once feel strong and don’t back down, that’s when she turns violent and starts to kick me. Yeah, that’s what she does. (M; G4)* |
|  |  | Self-sufficient | **He wouldn’t let you touch him, wouldn’t let you pick him up and hold him and comfort him. (F; B11)* |
|  |  |  | *He’s not shy, rather withdrawn. He doesn’t confide in grown-ups like that. I mean, I know when he has hurt himself. But he’s not one of those kids that go to the teachers for comfort. No, when he feels sad in school, he keeps to himself. He’ll go to the school’s backyard or something. (M; B1)* |
|  |  |  | *I’ve never been allowed to hug him or even touch him. He has never let me… I mean, just be close to him. (M; B10)* |
|  |  | Controlling | **Well, she screams, “You don’t love me. I’m not putting that sweater on because you don’t love me!” This can go on for an hour. If she doesn’t get what she wants, she can lie down on the floor and scream for an hour, basically until she falls asleep. (M; G9)* |
|  |  |  | *With me she can be… she’s controlling, she want to know 100 % what I‘m doing. Like now for example, I thought I’d tell her in the afternoon. I told her “ when daddy comes home I have to leave right away, I’m going to this old lady.” She goes “what are you gonna do there, what are you gonna do there?” She gets real… I notice at once how she gets anxious and distressed. Like “when will you be back again, when will you be back again?” This just goes on and on. And good-byes can be awfully tough and long sometimes. (M; G7)* |
|  |  |  | *So, you, know, it´s a power struggle between her and me. And I even feel sometimes in the morning, “I wonder how this morning is going to start out!” (M; G4)* |

*Means the quote is chosen to represent the parents´ different experience of unpredictably, aggressive, self-sufficient and controlling in the sub-theme of “Perceptions of the child´s negative behavior and emotions” in the manuscript.

** Total number of codes and quotes for one of the sub-theme in the bracket.
